# Antiamylase, Anticholinesterases, Antiglycation, and Glycation Reversing Potential of Bark and Leaf of Ceylon Cinnamon (*Cinnamomum zeylanicum* Blume) In Vitro

**DOI:** 10.1155/2017/5076029

**Published:** 2017-08-30

**Authors:** Sirimal Premakumara Galbada Arachchige, Walimuni Prabhashini Kaushalya Mendis Abeysekera, Wanigasekera Daya Ratnasooriya

**Affiliations:** ^1^Herbal Technology Section (HTS), Modern Research & Development Complex (MRDC), Industrial Technology Institute (ITI), 503A Halbarawa Gardens, Malabe, Sri Lanka; ^2^Department of Zoology, Faculty of Science, University of Colombo, Colombo, Sri Lanka; ^3^Faculty of Allied Health Sciences, General Sir John Kotelawala Defence University, Ratmalana, Sri Lanka

## Abstract

Ethanol (95%) and dichloromethane : methanol (DCM : M, 1 : 1 v/v) bark extracts (BEs) and leaf extracts (LEs) of authenticated Ceylon cinnamon (CC) were studied for antiamylase, antiglucosidase, anticholinesterases, and antiglycation and glycation reversing potential in bovine serum albumin- (BSA-) glucose and BSA-methylglyoxal models in vitro. Further, total proanthocyanidins (TP) were quantified. Results showed significant differences (*p* < 0.05) between bark and leaf extracts for the studied biological activities (except antiglucosidase) and TP. BEs showed significantly high (*p* < 0.05) activities for antiamylase (IC_50_: 214 ± 2–215 ± 10 *μ*g/mL), antibutyrylcholinesterase (IC_50_: 26.62 ± 1.66–36.09 ± 0.83 *μ*g/mL), and glycation reversing in BSA-glucose model (EC_50_: 94.33 ± 1.81–107.16 ± 3.95 *μ*g/mL) compared to LEs. In contrast, glycation reversing in BSA-methylglyoxal (EC_50_: ethanol: 122.15 ± 6.01 *μ*g/mL) and antiglycation in both BSA-glucose (IC_50_: ethanol: 15.22 ± 0.47 *μ*g/mL) and BSA-methylglyoxal models (IC_50_: DCM : M: 278.29 ± 8.55 *μ*g/mL) were significantly high (*p* < 0.05) in leaf. Compared to the reference drugs used some of the biological activities were significantly (*p* < 0.05) high (BEs: BChE inhibition and ethanol leaf: BSA-glucose mediated antiglycation), some were comparable (BEs: BSA-glucose mediated antiglycation), and some were moderate (BEs and LEs: antiamylase, AChE inhibition, and BSA-MGO mediated antiglycation; DCM : M leaf: BSA-glucose mediated antiglycation). TP were significantly high (*p* < 0.05) in BEs compared to LEs (BEs and LEs: 1097.90 ± 73.01–1381.53 ± 45.93 and 309.52 ± 2.81–434.24 ± 14.12 mg cyanidin equivalents/g extract, resp.). In conclusion, both bark and leaf of CC possess antidiabetic properties and thus may be useful in managing diabetes and its complications.

## 1. Introduction

Diabetes mellitus is one of the most prevalent chronic metabolic diseases worldwide [1, 2]. It affected about 387 million people worldwide in 2014 and the number is projected to increase by another 205 million people by 2035 [1]. The major categories of diabetes include type 1 and type 2 which is characterized by chronic hyperglycemia resulting from absolute or relative deficiencies in insulin secretion and/activity [2]. Prolonged hyperglycemic condition in diabetes patients induces nonenzymatic glycation reaction and leads to production of multitude of heterogeneous end products which are known as advanced glycation end products (AGEs) [3–7]. Low molecular weight carbonyl compounds such as glyoxal and methylglyoxal (MGO) behave as precursors of AGEs [5–8]. They form adducts on proteins, inducing cellular dysfunctions leading to long-term diabetes complications such as retinopathy, neuropathy, and nephropathy [3–7] and several age related diseases such as Alzheimer's disease, atherosclerosis, arthritis, pulmonary fibrosis, renal failure, and cancer [3–7]. Accumulation of AGEs in the brain is involved in extensive protein cross linking, oxidative stress, and neuronal cell death leading to neurodegenerative diseases and most commonly Alzheimer's disease [6, 9]. Currently Alzheimer's disease is even referred to as type 3 diabetes as it can be explained through AGEs and oxidation [9] and insulin and the cholinergic hypothesis [10, 11]. Natural products reported to have antidiabetic activity all over the world for centuries [12]. Antidiabetic drugs, nutraceuticals, and functional foods derived from plant sources have high demand as they are natural and safe alternatives to many synthetic drugs [13, 14]. Cinnamon, one of the oldest and most frequently consumed spices worldwide, belongs to the genus* Cinnamomum* and there are different species of cinnamon worldwide [15, 16]. Among several species of cinnamon in the world, Ceylon cinnamon is the “true cinnamon” the world over based on its unique taste, aroma, and phytochemical composition [15, 16]. Currently, Sri Lanka is the leading exporter of true cinnamon with 85% of world market share and 14.5% market share for all types of cinnamon worldwide. According to the recent statistics nearly 50% of export earnings of minor agricultural crops in the country come from Ceylon cinnamon [17].

Cinnamon is reported to have several pharmacological activities including some antidiabetic related properties worldwide [15, 16, 18, 26, 27]. However, main problem in many of these publications that there is no proper authentication for the experimental cinnamon sample [15]. Hence there is no strong evidence to confirm that these reported biological activities are from authenticated Ceylon cinnamon (true cinnamon) since the genus contains four economically important cinnamon species such as* Cinnamomum zeylanicum* or* Cinnamomum verum *(Ceylon cinnamon or true cinnamon),* Cinnamomum aromaticum* (*Cinnamomum cassia* or Chinese cinnamon),* Cinnamomum burmannii* (Korintje, Java, or Indonesian cinnamon), and* Cinnamomum loureiroi* (Vietnamese or Saigon cinnamon) [28]. On the other hand within the country there are no in-depth studies on antidiabetic activity of authenticated Ceylon cinnamon (except Ranasinghe et al. [29]) even though it is the most economical minor agricultural crop in Sri Lanka. Further, the studies conducted worldwide so far on antidiabetic activity of Ceylon cinnamon (true cinnamon) mainly focused on bark extracts and only 3 studies [20, 25, 30] are available on antidiabetic activity of leaf extracts to date. Further, as yet, there are no publish studies on antiamylase, antiglycation, and glycation reversing activities of bark and antiglucosidase, antiglycation, and glycation reversing potential of leaf of authenticated Ceylon cinnamon (true cinnamon) worldwide. Previous investigations on antiamylase, antiglucosidase, anticholinesterases, antiglycation, and glycation reversing activities of bark and leaf of* Cinnamomum* species are given in Table 1. The aim of this study was to evaluate antiamylase, antiglucosidase, anticholinesterases, antiglycation, and glycation reversing potential of both bark and leaf of authenticated Ceylon cinnamon via widely used, well established, sensitive, specific, reliable, and reproducible in vitro bioassays.

## 2. Materials and Methods

### 2.1. Chemicals and Reagents

Soluble starch, bovine serum albumin (BSA), D-glucose, *α*-glucosidase (type V from rice), p-nitrophenyl *α*-D-glucopyranoside, acarbose, trichloroacetic acid (TCA), acetylcholinesterase (AChE) from electric eel (Type-VI-S), butyrylcholinesterase (BChE) from horse serum, acetylthiocholine, butyrylthiocholine, 5,5′-dithio-bis-(2-nitrobenzoic) acid (DTNB), methylglyoxal (MGO), 3,5-dinitrosalicylic acid (DNS), dimethyl sulfoxide (DMSO), galantamine, rutin, cyanidin chloride, and ammonium iron(III) sulfate dodecahydrate were purchased from Sigma-Aldrich, USA. *α*-Amylase* (Bacillus amyloliquefaciens)* was purchased from Roche Diagnostics, USA. All the other chemicals and reagents were of analytical grade.

### 2.2. Collection and Preparation of Ceylon Cinnamon Alba Grade Bark and Leaf Samples

Fresh cinnamon leaves were collected from cinnamon cultivations of L.B. spices (Pvt) Ltd., Aluthwala, Galle, Sri Lanka. Alba grade cinnamon bark samples (alba grade cinnamon has the lowest quill thickness, maximum 6 mm, according to the grading of cinnamon quills based on the quill thickness) [31] were collected from cinnamon factories of L.B. spices (Pvt) Ltd., Aluthwala, Galle, Sri Lanka, and G. P. De Silva and Sons Spice (Pvt) Ltd., Ambalangoda, Sri Lanka. The alba grade bark samples were authenticated by Dr. Chandima Wijesiriwardena, Principle Research Scientist, Industrial Technology Institute, Sri Lanka, and leaf samples (voucher number CZB-KA) were authenticated by Mr. N.P.T. Gunawardena, Officer In-Charge, National Herbarium, Department of National Botanic Gardens, Peradeniya, Sri Lanka. The specimens of each bark and leaf samples (HTS-CIN-1) and photographic evidence were deposited at the Pharmacognosy Laboratory, Herbal Technology Section, Industrial Technology Institute, Sri Lanka. Fresh leaves were air-dried at room temperature (30 ± 2°C) for 7 days. The air-dried leaves and bark were ground, powdered, and stored at −20°C until used for the extraction.

### 2.3. Preparation of Extracts

#### 2.3.1. Preparation of Ethanolic Extracts

Powdered bark and leaf samples (20 g) were extracted in 200 mL of 95% ethanol for 4-5 h in a Soxhlet extractor (4–6 cycles) until the solvent became colorless. The extracts were filtered, evaporated, and freeze-dried (Christ-Alpha 1–4 Freeze dryer, Biotech International, Germany). Freeze-dried extracts were stored at −20°C until used for analysis.

#### 2.3.2. Preparation of Dichloromethane : Methanol (DCM : M) Extracts

Powdered bark and leaf (20 g) samples were extracted in 200 mL of dichloromethane : methanol (DCM : M) at a ratio of (1 : 1, v/v) at room temperature (30 ± 2°C) for 7 days with occasional shaking. The extracts were filtered, evaporated, freeze-dried, and stored at −20°C until used for analysis.

### 2.4. Antiamylase Activity

The antiamylase activity of bark and leaf extracts of Ceylon cinnamon were carried out according to the method of Bernfeld [32] with some modifications. Briefly, a reaction volume of 1 mL containing 50 *μ*L of ethanolic and DCM : M bark and leaf extracts (bark extracts: 62.5, 125, 250, 500, and 1000 *μ*g/mL, *n* = 4; leaf extracts: 93.75, 187.50, 375, 750, and 1500 *μ*g/mL, *n* = 4), 40 *μ*L of starch (1%, w/v), and 50 *μ*L of enzyme (5 *μ*g/mL) in 100 mM sodium acetate buffer (pH 6.0) were incubated at 40°C for 15 min. After the incubation period, 0.5 mL of DNS reagent was added and placed in a boiling water bath for 5 min. Then reaction mixtures were cooled in a water bath containing ice and absorbance readings were recorded at 540 nm using a 96-well microplate reader (SpectraMax PLUS 384, Molecular Devices, Inc., USA). Control of the experiment contains all the reagents except extracts, whereas sample blanks were without the enzyme. Acarbose was used as the positive control (6.25–100 *μ*g/mL). Antiamylase activity (% inhibition) was given as IC_50_ values (concentration of bark and leaf extracts and positive control that inhibited the hydrolysis of starch by 50%). Inhibition % was calculated using the following:

(1) $$\begin{array}{c} \text{Inhibition}\;\left(\;\%\;\right)=\left[\;\frac{A_{c}-\left(A_{s}-A_{b}\right)}{A_{c}}\;\right]*100, \end{array}$$

where *A*_*c*_ is the absorbance of the control, *A*_*b*_ is the absorbance of sample blanks, and *A*_*s*_ is the absorbance in the presence of bark and leaf extracts.

### 2.5. Antiglucosidase Activity

Antiglucosidase activity of bark and leaf extracts of Ceylon cinnamon was carried out according to the method of Matsui et al. [33] with minor modifications in 96-well microplates. A reaction volume of 0.1 mL containing 4 mM p-nitrophenyl-*α*-D-glucopyranoside, 50 mU/mL of *α*-glucosidase, and 40 *μ*L of ethanolic and DCM : M bark and leaf extracts (25, 50, 100, 200, and 400 *μ*g/mL; *n* = 4) in 50 mM sodium acetate buffer (pH 5.8) were incubated at 37°C for 30 min. After the incubation period, reaction was stopped by adding 50 *μ*L of 0.1 M Na_2_CO_3_. Then, absorbance readings were taken at 405 nm using a 96-well microplate reader. Reaction mixture without extract was used as the control and reaction mixture with the extract and without enzyme was used as the sample blank. Acarbose, a clinical *α*-glucosidase inhibitor, was used as the positive control. Antiglucosidase activity (% inhibition) was calculated by using the following:

(2) $$\begin{array}{c} \text{Inhibition}\;\left(\;\%\;\right)=\left[\;\frac{A_{c}-\left(A_{s}-A_{b}\right)}{A_{c}}\;\right]*100, \end{array}$$

where *A*_*c*_ is the absorbance of the control (100% enzyme activity), *A*_*b*_ is the absorbance produced by cinnamon extracts (sample blank), and *A*_*s*_ is the absorbance of the sample in the presence of cinnamon bark or leaf extracts or acarbose.

### 2.6. Anticholinesterase Activity

AChE and BChE inhibitory activities of bark and leaf extracts of Ceylon cinnamon were performed according to the method of Ellman et al. [34] with some modifications in 96-well microplates. A reaction volume of 200 *μ*L containing 0.1 M sodium phosphate buffer (pH 8.0), 15/0.03 mU of AChE/BChE (10 *μ*L) enzyme and 50 *μ*L of different concentrations of bark and leaf extracts (both bark and leaf for AChE: 50, 100, 200, 400, and 800 *μ*g/mL; bark BChE: 6.25, 12.5, 25, 50, and 100 *μ*g/mL; leaf BChE: 25, 50, 100, 200, and 400 *μ*g/mL) and the positive controls were preincubated for 15 min at 25°C. The reaction was then initiated by the addition of 10/20 *μ*L of 2 mM acetylthiocholine/butyrylthiocholine and 20 *μ*L of 0.5 mM DNTB. The hydrolysis of acetylthiocholine/butyrylthiocholine was monitored by the formation of yellow colored 5-thio-2-nitrobenzoate anion for a period of 10 min for BChE and 20 min for AChE at 412 nm using 96-well microplate reader (SpectraMax Plus^384^, Molecular Devices, USA). Galantamine was used as the positive control (AChE 0.39–25 *μ*g/mL; BChE 12.5–200 *μ*g/mL). Control incubations were carried out in the same way while replacing extracts with buffer. The kinetic parameter *V*_max_ was used to calculate the % inhibition and anticholinesterase activity was given as IC_50_ values (the concentrations of bark and leaf extracts and the positive control that inhibited the hydrolysis of acetylcholine/butyrylthiocholine by 50%).

The percentage inhibition was calculated as

(3) $$\begin{array}{c} \text{Inhibition}\;\left(\;\%\;\right)=\frac{\left(A_{C}-A_{S}\right)}{A_{C}}\times 100, \end{array}$$

where *A*_*C*_ is *V*_max_ of the control and *A*_*S*_ is *V*_max_ of the sample or galantamine.

### 2.7. Antiglycation Activity

#### 2.7.1. BSA-Glucose Glycation Inhibitory Activity

This assay was carried out according to the method of Matsuura et al. [35] with some modifications. A reaction mixture of 1 mL containing 800 *μ*g BSA, 400 mM glucose, and 50 *μ*L of ethanolic and DCM : M bark and leaf extracts (6.25, 12.5, 25, 50, 75, and 100 *μ*g/mL; *n* = 4) in 50 mM phosphate buffer (pH 7.4) containing 0.02% sodium azide were incubated for 40 h at 60°C. The 600 *μ*L of each reaction mixture was transferred to 1.5 mL Eppendorf tubes and 60 *μ*L of 100% (w/v) TCA was added, mixed well, and allowed to stand at room temperature (25 ± 2°C) for 30 min. Then sample mixtures were centrifuged at 15,000 rpm at 4°C for 4 min and supernatants were discarded. The AGEs-BSA precipitate was then dissolved in 1 mL of phosphate buffer saline (pH 10) and the fluorescence intensity was measured at an excitation and emission wave lengths of 370 nm and 440 nm using a 96-well florescence microplate reader (SpectraMax, Gemini EM, Molecular Devices, Inc., USA). Rutin was used as the positive control (6.25–100 *μ*g/mL). Antiglycation activity (% inhibition) was calculated using the following equation. IC_50_ values (concentration of bark and leaf extracts and rutin that inhibited the formation of AGEs by 50%) were also calculated:

(4) $$\begin{array}{c} \text{Inhibition}\;\left(\;\%\;\right)=\left[\;\frac{\left(F_{c}-F_{b}\right)-\left(F_{s}-F_{sb}\right)}{\left(F_{c}-F_{b}\right)}\;\right]*100, \end{array}$$

where *F*_*c*_ is the florescence of incubated BSA, glucose, and DMSO (control), *F*_*b*_ is the florescence of incubated BSA alone (blank), *F*_*s*_ is the florescence of the incubated BSA, glucose, and cinnamon leaf or bark extracts or the positive control, and *F*_*sb*_ is the florescence of incubated BSA with the leaf or bark extracts or the positive control.

#### 2.7.2. BSA-MGO Glycation Inhibitory Activity

This assay was carried out according to the method reported by Lunceford and Gugliucci [36] with some modifications. Reaction volume of 1 mL containing 1 mg BSA, 5 mM MGO, and different concentrations of ethanolic and DCM : M bark and leaf extracts (25, 50, 100, 200, and 400 *μ*g/mL; *n* = 6) in 0.1 M phosphate buffer containing 0.2 g/L sodium azide were incubated at 37°C for 6 days. After the incubation period, florescence was measured at an excitation and emission wavelengths of 370 and 440 nm using 96-well florescence microplate reader. Control experiments were conducted in an identical way while replacing extracts with 0.1 M phosphate buffer. For sample blanks, MGO solution was replaced with 0.1 M phosphate buffer. Rutin was used as the positive control (6.25–200 *μ*g/mL). Antiglycation activity (inhibition %) was calculated as described in BSA/glucose model by replacing glucose with MGO.

### 2.8. Glycation Reversing Activity

#### 2.8.1. BSA-Glucose Glycation Reversing Activity

This assay was carried out according to the method of Premakumara et al. [37] with some modifications. A reaction mixture containing 800 *μ*g BSA and 400 mM glucose in 1 mL of 50 mM phosphate buffer (pH 7.4) containing 0.02% sodium aside (w/v) was incubated at 60°C for 40 h. Then 600 *μ*L of each reaction mixtures was transferred to 1.5 mL Eppendorf tubes and 60 *μ*L of 100% (w/v) TCA was added, stirred well, and allowed to stand at room temperature for 30 min. Then, sample mixtures were centrifuged at 15,000 rpm at 4°C for 4 min and supernatants were discarded. The resulting AGEs-BSA precipitates were dissolved in 50 mM phosphate buffer (pH 7.4) added with 12.5, 25, 50, 100, 150, and 200 *μ*g/mL bark and leaf extracts (*n* = 6) in a final reaction volume of 1 mL and were incubated at 60°C for 40 h. After cooling, 60 *μ*L of 100% (w/v) TCA was added, stirred, and centrifuged at 15,000 rpm at 4°C for 4 min. The resulting precipitates were then dissolved in 1 mL of phosphate buffer saline (pH 10) and fluorescence intensity was measured at an excitation wave length of 370 nm and emission wave length of 440 nm using a 96-well florescence microplate reader. Percentage glycation reversing was calculated using the following equation and results were given as EC_50_ values (concentration of bark and leaf extracts that reversed the AGEs by 50%):

(5) Glycation  reversing %=Fc−Fb−Fs−FsbFc−Fb∗100,

where *F*_*c*_ is the florescence of incubated BSA, glucose, and DMSO (control), *F*_*b*_ is the florescence of incubated BSA alone (blank), *F*_*s*_ is the florescence of the incubated BSA, glucose, and bark/leaf extracts, and *F*_*sb*_ is the florescence of incubated BSA with the bark/leaf extracts.

#### 2.8.2. BSA-MGO Glycation Reversing Activity

This assay was performed according to the method of Lunceford and Gugliucci [36] and Premakumara et al. [37] with minor modifications. Reaction mixture containing 1 mg BSA and 5 mM MGO in 1 mL of 0.1 M phosphate buffer pH 7.4 was incubated at 37°C for 6 days. The test solution also contained 0.2 g/L NaN_3_ to assure an aseptic condition. Then, aliquots of 600 *μ*L were transferred to 1.5 mL Eppendorf tubes and 60 *μ*L of 100% (w/v) TCA was added, stirred, and centrifuged at 15,000 rpm at 4°C for 4 min and supernatants were removed. The resulting precipitates were dissolved in 0.1 M phosphate buffer (pH 7.4) and added with 37.5, 75, 150, 300, and 600 *μ*g/mL (*n* = 4) bark and leaf extracts to a final reaction volume of 1 mL for incubation at 37°C for 6 days. After the incubation, florescence was measured at an excitation wave length of 370 nm and emission wave length of 440 nm using a 96-well florescence microplate reader. Percentage glycation reversing was calculated as described in BSA/glucose reversing model via replacing glucose with MGO.

### 2.9. Total Proanthocyanidin Content

The total proanthocyanidin content of bark and leaf extracts of Ceylon cinnamon was quantified by butanol-HCl assay method described by Porter et al. [38] with minor modifications. Reaction volumes of 3.6 mL containing 0.5 mL of extracts in methanol (assay concentration: ethanolic and DCM : M bark and leaf extracts: 0.25 mg/mL, *n* = 6 each), 3 mL of butanol-HCl reagent (95 : 5, v/v), and 100 *μ*L of 2% ammonium iron(III) sulfate dodecahydrate in 2 M HCl were added to 10 mL screw capped test tubes, mixed well, and incubated at 95°C in a water bath for 40 min. Sample blanks were carried out in the same way without heating. After the incubation period, samples were allowed to cool to room temperature and absorbance was recorded at 550 nm. Cyanidin chloride (0.016, 0.031, 0.063, 0.125, and 0.25 mg/mL; *n* = 3) was used as the standard. Results were expressed as mg cyanidin equivalents per g of extract of cinnamon bark/leaf.

### 2.10. Statistical Analysis

Data of each experiment were statistically analyzed using SAS version 6.12. One way analysis of variance (ANOVA) and the Duncan's Multiple Range Test (DMRT) were used to determine the differences among treatment means. *p* < 0.05 was regarded as significant.

## 3. Results

### 3.1. Antiamylase Activity of Bark and Leaf Extracts of Ceylon Cinnamon

Both bark and leaf extracts demonstrated antiamylase activity in a dose-dependent manner (ethanol bark, DCM : M bark, ethanol leaf, DCM : M leaf *r*^2^ = 0.99, 1.00, 1.00, and 0.95, resp.). However, bark extracts showed significantly higher activity (*p* < 0.05) compared to leaf extracts. Antiamylase activity between ethanol and DCM : M bark extracts were statistically non-significant (*p* > 0.05). The IC_50_ values of ethanolic bark and DCM : M bark were 215 ± 10 and 214 ± 2 *μ*g/mL, respectively. Among the studied leaf extracts, ethanolic leaf extract had high antiamylase activity (IC_50_943 ± 28 *μ*g/mL) than DCM : M leaf extract (17.59 ± 1.24% inhibition at 1.5 mg/mL). Further, both bark and leaf extracts showed moderate antiamylase activity compared to the standard drug acarbose (IC_50_133.88 ± 2.54 *μ*g/mL). The dose-response relationship of bark and leaf extracts for antiamylase activity is given in Table 2.

### 3.2. Antiglucosidase Activity of Bark and Leaf Extracts of Ceylon Cinnamon

Both ethanolic and DCM : M bark and leaf extracts did not show antiglucosidase activity even at the highest studied concentration of 400 *μ*g/mL. Results of antiglucosidase activity of bark and leaf extracts were given in Table 3. Acarbose, a clinical *α*-glucosidase inhibitor, had antiglucosidase activity as IC_50_ = 0.47 ± 0.01 *μ*g/mL.

### 3.3. Anticholinesterase Activity of Bark and Leaf Extracts of Ceylon Cinnamon

Both ethanolic and DCM : M bark and leaf extracts of Ceylon cinnamon showed both AChE and BChE inhibitory activities. However, inhibition of BChE was more prominent compared to AChE inhibition in both bark and leaf extracts. Bark extracts showed dose-dependent (ethanol bark and DCM : M bark *r*^2^ = 0.97 each) and significantly high (ethanol bark and DCM : M bark IC_50_  36.09 ± 0.83 and 26.62 ± 1.66 *μ*g/mL, resp.) (*p* < 0.05) BChE inhibition compared to the standard drug galantamine (IC_50_  74.80 ± 3.53 *μ*g/mL). On the other hand, BChE inhibition of leaf extracts although dose-dependent (ethanol leaf and DCM : M leaf *r*^2^ = 0.94 and 0.98, resp.) was moderate (ethanol leaf and DCM : M leaf: IC_50_: 340.60 ± 18.23 and 261.96 ± 19.56 *μ*g/mL, resp.). Further, DCM : M extracts showed significantly high (*p* < 0.05) activity than ethanol extracts in both bark and leaf. In complete contrast, AChE inhibition of bark and leaf extracts showed dose-dependent (ethanol bark, DCM : M bark, ethanol leaf, and DCM : M leaf *r*^2^ = 0.92, 0.94, 0.95, and 0.99, resp.) but significantly low (*p* < 0.05) activity with respect to standard drug galantamine. The IC_50_ values of ethanol bark, DCM : M bark, ethanol leaf, DCM : M leaf, and galantamine were 804.88 ± 48.69, 966.68 ± 63.18, 810.96 ± 79.98, 879.35 ± 68.00, and 2.52 ± 0.17 *μ*g/mL, respectively. The dose-response relationships of ethanol and DCM : M bark and leaf extracts for acetyl and butyrylcholinesterase inhibitory activities are given in Table 4.

### 3.4. Antiglycation Potential of Bark and Leaf Extracts of Ceylon Cinnamon

#### 3.4.1. BSA-Glucose Glycation Inhibitory Activity

**Figure 1:**
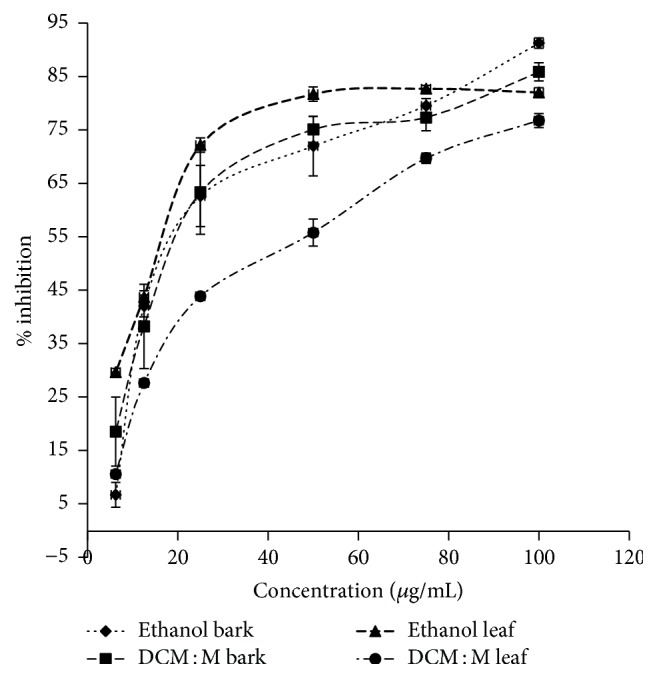
Antiglycation activity via BSA-glucose model. Data represented as mean ± SEM (*n* = 4 each). IC_50_ values: ethanol leaf, ethanol bark, DCM : M bark, and DCM : M leaf: 15.22 ± 0.47^c^, 19.42 ± 1.26^b^, 20.80 ± 2.68^b^, and 42.62 ± 1.67^a^ g/mL, respectively. IC_50_ values superscripted by different letters are significantly different at *p* < 0.05. Ethanol leaf, ethanol bark, DCM : M bark, and DCM : M leaf: *r*^2^ = 1.00, 0.89, 0.99, and 0.96, respectively. IC_50_: rutin: 21.88 ± 2.82 *μ*g/mL. DCM : M: dichloromethane : methanol.

Both ethanolic and DCM : M bark and leaf extracts showed dose-dependent antiglycation activity (ethanol bark, DCM : M bark, ethanol leaf, and DCM : M leaf *r*^2^ = 0.89, 0.99, 1.00, and 0.96, resp.). IC_50_ values of bark and leaf extracts ranged from 19.42 ± 1.26–20.80 ± 2.68 to 15.22 ± 0.47–42.62 ± 1.67 *μ*g/mL, respectively. Ethanol leaf had the highest BSA-glucose glycation inhibitory activity (IC_50_  15.22 ± 0.47 *μ*g/mL). Further, both bark extracts showed similar (ethanol and DCM : M bark extracts: IC_50_  19.42 ± 1.26 and 20.80 ± 2.68 *μ*g/mL, resp.) and DCM : M leaf showed lowest antiglycation activity (IC_50_  42.62 ± 1.67 *μ*g/mL). Antiglycation activity of ethanolic leaf and bark extracts was significantly higher (*p* < 0.05) and comparable compared to the positive control, rutin (IC_50_  21.88 ± 2.82 *μ*g/mL). Dose-response relationships of ethanolic and DCM : M bark and leaf extracts of Ceylon cinnamon are given in Figure 1.

#### 3.4.2. BSA-MGO Glycation Inhibitory Activity

**Figure 2:**
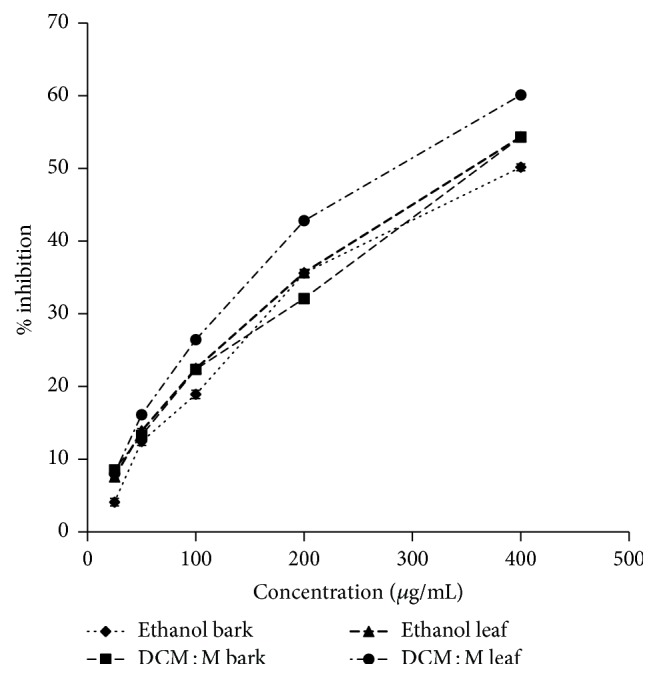
Antiglycation activity via BSA-MGO model. Data represented as mean ± SEM (*n* = 4 each). IC_50_ values: DCM : M leaf, ethanol leaf, DCM : M bark, and ethanol bark: 278.29 ± 8.55^c^, 349.28 ± 8.21^b^, 357.38 ± 3.08^b^, and 392.59 ± 20.88^a^ *μ*g/mL, respectively. IC_50_ values superscripted by different letters are significantly different at *p* < 0.05. DCM : M leaf, ethanol leaf, DCM : M bark, and ethanol bark *r*^2^ = 0.95, 0.98, 0.99, and 0.95 respectively. IC_50_: rutin: 63.35 ± 0.67 *μ*g/mL. DCM : M: dichloromethane : methanol; Methylgloxal: MGO.

Both bark and leaf extracts of Ceylon cinnamon showed BSA-MGO glycation inhibitory activity. The inhibitory activity of BSA-MGO glycation was dose-dependent (ethanol bark, DCM : M bark, ethanol leaf, and DCM : M leaf *r*^2^ = 0.95, 0.99, 0.98, and 0.95, resp.) and moderate compared to the standard drug rutin (IC_50_  63.35 ± 0.67 *μ*g/mL). The IC_50_ values of bark and leaf extracts ranged from 357.38 ± 3.08–392.59 ± 20.88 to 278.29 ± 8.55–349.28 ± 8.21 *μ*g/mL, respectively. DCM : M extracts of both bark and leaf showed significantly (*p* < 0.05) high activity compared to ethanol extracts. The order of potency of BSA-MGO glycation inhibitory activity was DCM : M leaf > ethanol leaf = DCM : M bark > ethanol bark. Dose-response relationships of ethanolic and DCM : M bark and leaf extracts of Ceylon cinnamon are given in Figure 2.

### 3.5. Glycation Reversing Activity

#### 3.5.1. BSA-Glucose Glycation Reversing Activity

**Figure 3:**
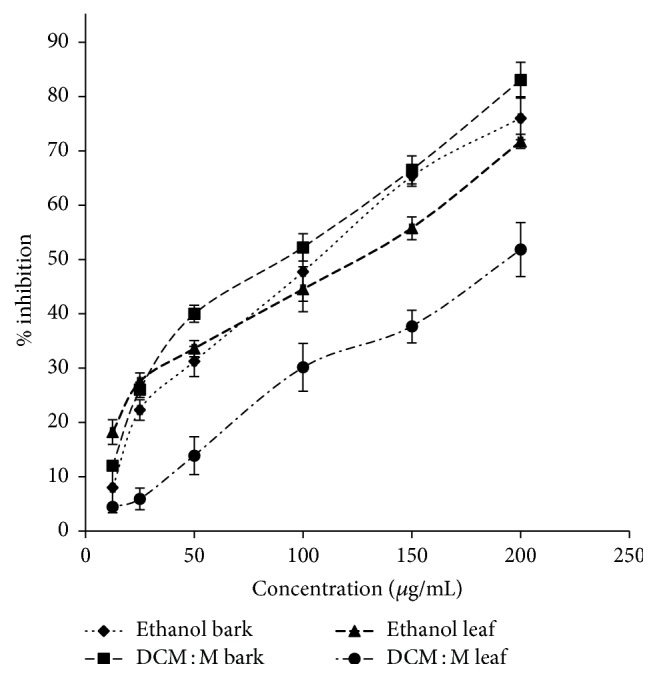
Glycation reversing activity via BSA-glucose model. Data represented as mean ± SEM (*n* = 6 each). EC_50_ values: DCM : M bark, ethanol bark, ethanol leaf, and DCM : M leaf: 94.33 ± 1.81^d^, 107.16 ± 3.95^c^, 121.20 ± 2.01^b^, and 199.42 ± 9.02^a^ *μ*g/mL, respectively. EC_50_ values superscripted by different letters are significantly different at *p* < 0.05. DCM : M bark, ethanol bark, ethanol leaf, and DCM : M leaf *r*^2^ = 0.96, 0.97, 0.99, and 0.99, respectively. DCM : M: dichloromethane : methanol.

Both bark and leaf extracts showed significant and dose-dependent (ethanol bark, DCM : M bark, ethanol leaf, and DCM : M leaf *r*^2^ = 0.97, 0.96, 0.99, and 0.99, resp.) BSA-glucose glycation reversing activity. IC_50_ values of bark and leaf extracts ranged from 94.33 ± 1.81–107.16 ± 3.95 to 121.20 ± 2.01–199.42 ± 9.02 *μ*g/mL, respectively. Bark extracts showed significantly high activity than leaf extracts (*p* < 0.05). The order of potency of BSA-glucose glycation reversing activity was DCM : M bark > ethanol bark > ethanol leaf > DCM : M leaf. Dose-response relationships of ethanolic and DCM : M bark and leaf extracts of Ceylon cinnamon are given in Figure 3.

#### 3.5.2. BSA-MGO Glycation Reversing Activity

**Figure 4:**
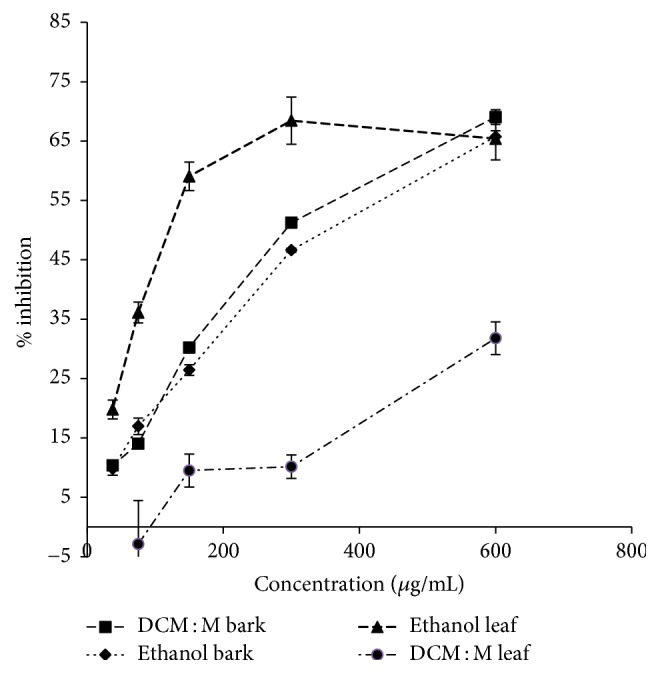
Glycation reversing activity via BSA-MGO model. Data represented as mean ± SEM (*n* = 4 each). EC_50_ values: ethanol leaf, DCM : M bark, and ethanol bark: 122.15 ± 6.01^c^, 287.80 ± 3.20^b^, and 322.83 ± 1.76^a^ *μ*g/mL, respectively. EC_50_ values superscripted by different letters are significantly different at *p* < 0.05. Ethanol leaf, ethanol bark, and DCM : M bark *r*^2^ = 0.99, 0.94, and 0.96, respectively. DCM : M: dichloromethane : methanol; Methylgloxal: MGO.

Both ethanolic and DCM : M bark and leaf extracts of Ceylon cinnamon showed dose-dependent (ethanol bark, DCM : M bark, ethanol leaf and DCM : M leaf *r*^2^ = 0.94, 0.96, 0.99, and 0.90, resp.) BSA-MGO glycation reversing activity. However, ethanolic leaf extract showed the highest reversing ability while DCM : M extract of leaf showed the lowest reversing activity. The order of potency of BSA-MGO glycation reversing activity was ethanol leaf > DCM : M bark > ethanol bark > DCM : M leaf. Dose-response relationships of ethanolic and DCM : M bark and leaf extracts of Ceylon cinnamon for BSA-MGO glycation reversing are given in Figure 4.

### 3.6. Total Proanthocyanidin Content

Total proanthocyanidin content of ethanolic and DCM : M bark and leaf extracts of Ceylon cinnamon is given in Table 5. Mean total proanthocyanidin content of bark and leaf extracts of cinnamon ranged from 309.52 ± 2.81 to 1381.53 ± 45.93 mg cyanidin equivalents/g extract. Both bark extracts had significantly high total proanthocyanidin content (1097.90 ± 73.01–1381.53 ± 45.93 mg cyanidin equivalents/g extract) than both leaf extracts (309.52 ± 2.81–434.24 ± 14.12 mg cyanidin equivalents/g extract) (*p* < 0.05). The order of mean total proanthocyanidin content was DCM : M bark > ethanol bark > ethanol leaf > DCM : M leaf.

## 4. Discussion

A range of selected antidiabetic properties [antiamylase, antiglucosidase, anticholinesterases, antiglycation, and glycation reversing activities] of alba grade bark and leaf of Ceylon cinnamon were evaluated using well established, widely used, sensitive, specific, validated, and internationally accepted antidiabetic bioassays in vitro [32–37]. Alba grade bark of Ceylon cinnamon was used since which is the most highly priced cinnamon grade in the international trade (due to its finest quill thickness, unique aroma, and taste). Leaf extracts were also evaluated for antidiabetic related properties as leaf is claimed to have antidiabetic activity in Sri Lankan traditional knowledge [39] and folklore. Ethanol and DCM : M bark and leaf extracts were used as these extracts have been previously used in the investigation of antioxidants and antioxidant activity [40] and antilipidemic activity in vitro [16].

*α*-Amylase and *α*-glucosidases are the key enzymes involved in starch digestion process [18]. Thus, inhibitors of these enzymes can play a key role in the management of diabetes. Both bark (IC_50_: 214 ± 2–215 ± 10 *μ*g/mL) and leaf (IC_50_: 943 ± 28 *μ*g/mL) of Ceylon cinnamon showed antiamylase activity. Antiamylase activity of both bark extracts was significantly high compared to both leaf extracts while it was moderate compared to the reference drug acarbose (IC_50_: 133.88 ± 4.4 *μ*g/mL). Previous investigation on *α*-amylase inhibitory activity of bark of some economically important* Cinnamomum* species such as* C. zeylanicum*,* C. aromaticum*, and* C. loureiroi* showed that it had antiamylase activity and activity as IC_50_ values 1.23 ± 0.02, 1.77 ± 0.05, and >4.00 mg/mL, respectively [18]. According to the above research bark of* C. zeylanicum *had the highest antiamylase activity among the studied economically important* Cinnamomum* species. Further, Beejmohun et al. [19] reported antiamylase activity of bark of* C. zeylanicum *as IC_50_ value 25 *μ*g/mL. Compared to the above studies antiamylase activity of bark of Ceylon cinnamon was ranging from 6 times higher to 25 times lower in the present study. The present study was conducted using ethanolic and DCM : M extracts of authenticated bark of Ceylon cinnamon and* Bacillus amyloliquefaciensα*-amylase as the source of amylase. On the other hand the studies conducted by Adisakwattana et al. [18] and Beejmohun et al. [19] were used water and hydroalcoholic extracts of bark and porcine pancreatic *α*-amylase as the source of amylase. Further, the cinnamon samples used in both studies were not authenticated. Therefore, discrepancy observed between present study and previous investigations on antiamylase activity may be due to the use of different solvents, extraction procedures, source of *α*-amylase, and use of cinnamon samples without proper authentication. Compared to bark, leaf of* C. zeylanicum (C. verum) *was rarely investigated for antiamylase activity to date. Research carried out by Ponnusamy et al. [20] reported that antiamylase activity of leaf extract of* C. verum* as IC_50_ value 1 *μ*g/mL. Compared to above study, *α*-amylase inhibitory activity of leaf of Ceylon cinnamon was nearly 950 times lower in the present study. Isopropanol leaf extract and human pancreatic *α*-amylase were used by Ponnusamy et al. [20] to evaluate the antiamylase activity of leaf of* C. verum*. Therefore, discrepancy observed between present study and study conducted by Ponnusamy et al. [20] on *α*-amylase inhibitory activity of leaf may be due to the use of different solvents, extraction procedures, source of *α*-amylase, and use of cinnamon samples without proper authentication. To the best of our knowledge except our research this is the only available report on antiamylase activity of leaf of any* Cinnamomum* species worldwide.

A recent study has shown that* C. zeylanicum* bark possesses *α*-glucosidase inhibitory activity [21]. However, both bark and leaf extracts of Ceylon cinnamon did not show *α*-glucosidase inhibition in the present study at studied concentrations. The inhibitory activity observed by Ranilla et al. [21] was at high concentrations (100% inhibition at 2.5 mg/mL and 95% inhibition at 0.5 mg/mL) and it is beyond the maximum concentration (400 *μ*g/mL) used in our experiments. Nevertheless, some recent studies have shown that Ceylon cinnamon bark has intestinal maltase and sucrase inhibitory activities [15, 18]. Besides the bark, except our research on antiglucosidase activity of leaf of Ceylon cinnamon none of the leaf extracts of* Cinnamomum* species reported to have antiglucosidase activity to date. However, ability to impair postprandial intestinal glucose absorption by inhibiting the activity of enzymes involved in carbohydrate metabolism (*α*–amylase and *α*–glucosidase) by both bark [15, 18, 21] and leaf of Ceylon cinnamon in the present and previous studies indicates its potential use as food supplements, nutraceuticals, and functional foods in the management of diabetes and related complications.

Prolonged hyperglycemic condition in diabetes patients induces formation of AGEs which are positively correlated with development and progression of several diabetes complications and age related diseases [3–7]. The process of AGEs formation through protein glycation is not a single step reaction [4, 6]. This reaction can be broadly divided into three stages as early, middle, and late stage glycation [41]. In this study inhibitory activity of Ceylon cinnamon on protein glycation in BSA-glucose and BSA-MGO models represents early and middle stages of protein glycation process, respectively [36]. Reversing of already formed AGEs and/or cross link braking is another vital approach for attenuation of AGEs related complications [6, 42]. To date very few compounds are known to have AGE cross links breaking capacity. 1,3-Thiazolium derivatives, such as N-phenyl-1,3-thiazolium bromide (PTB) and N-phenacyl-4,5-dimethyl-1,3-thiazolium chloride (alagebrium chloride), are important protein crosslink breakers. However, these compounds reported to have limited efficacy in in vivo studies [6, 42]. Therefore, discovery of inhibitors which can inhibit all stages of glycation process and reversing of already formed AGEs would offer a potential therapeutic approach for the prevention of diabetes complications and AGEs related pathologies.

The results demonstrated for the first time that both bark and leaf of authenticated true Ceylon cinnamon can inhibit both early and middle stages of protein glycation process. Inhibition of early stage protein glycation was more potent (ethanolic leaf extract IC_50_: 15.22 ± 0.47 *μ*g/mL) or comparable (bark extracts IC_50_: 19.42 ± 1.26–20.80 ± 2.68 *μ*g/mL) to the reference standard rutin (IC_50_: 21.88 ± 2.82 *μ*g/mL) while it was moderate in middle stage protein glycation in both bark and leaf extracts. Previous investigation on antiglycation activity of cinnamon by Peng et al. [22] reported that different fractions including ethyl acetate and 1-butanol fractions of water extract of bark of cinnamon had BSA-glucose glycation inhibition with 66.2 and 59.5%, respectively, at a concentration of 200 ppm. Further, Ho and Chang [23] reported that IC_50_ value of BSA-glucose glycation inhibitory activity of cinnamon as 26 *μ*g/mL. Compared to the above studies the BSA-glucose glycation inhibitory activity of bark of authenticated Ceylon cinnamon in the present study is similar to IC_50_ value reported by Ho and Chang [23]. However, the cinnamon samples used in both the studies were not authenticated and hence contradictory about the cinnamon species used in those experiments. On the other hand antiglycation activity of leaf of* C. zeylanicum* is not reported to date. Therefore, this is the first study to report that both bark and leaf extracts of authenticated true Ceylon cinnamon can inhibit both early and middle stages of protein glycation process. Further, both bark and leaf extracts exhibited the glycation reversing ability in both BSA-glucose and BSA-MGO glycated products and this is the first report of glycated products reversing ability of any* Cinnamomum* species worldwide. The presence of antiglycation potential of bark and leaf of Ceylon cinnamon indicates its ability to ameliorate various diabetes and age related complications.

Alzheimer's disease is characterized by inadequate production of acetylcholine in the brain and recently it is referred as type 3 diabetes as insulin plays a significant role in the expression of choline acetyltransferase, the enzyme responsible for the synthesis of acetylcholine [10, 11]. Both bark and leaf of Ceylon cinnamon showed moderate AChE (IC_50_: 804.88 ± 48.69–966.68 ± 63.1 *μ*g/mL) and moderate to high BChE inhibitory activities (IC_50_: 26.62 ± 1.66–340.60 ± 18.23 *μ*g/mL) compared to the reference drug galantamine (IC_50_: AChE 2.52 ± 0.17 *μ*g/mL; BChE: 74.80 ± 3.53 *μ*g/mL). Both AChE and BChE play an important role in cholinergic signaling. A recent research has shown that reduction of AChE activity can be compensated by increasing BChE activity since BChE can even hydrolyze acetylcholine when AChE levels are depleted in Alzheimer's patients [43, 44]. Therefore, currently BChE inhibitors such as cymserine analogues and the dual inhibitor of both AChE and BChE such as rivastigmine are used therapeutically for treating Alzheimer's disease and other related dementias [43]. As Ceylon cinnamon bark and leaf demonstrated both AChE and BChE inhibitory activities consumption in daily life could increase the acetylcholine level and would be beneficial for management of Alzheimer's disease. This is the first report of AChE and BChE inhibitory activities of authenticated leaf of Ceylon cinnamon worldwide.

Several research studies have clearly shown that oxidative stress plays a key role in pathological processes observed in diabetes mellitus [3–7]. The use of antioxidant therapy has shown beneficial effects for management of pathologies associated with oxidative stress in diabetes patients [45]. Further, several researches have shown that antioxidants including phenolic compounds play an important role in mediating antiglycation activity and inhibitory activity towards amylase and cholinesterase enzymes [37, 46]. In our previous study Ceylon cinnamon bark and leaf extracts were shown to have high antioxidant activity and phenolic contents [40]. Further, in the present study both bark extracts showed high and both leaf extracts showed moderate total proanthocyanidins content. Therefore, the observed antidiabetic related properties of Ceylon cinnamon may be attributed, at least partly, to phenolic compounds including proanthocyanidins and other antioxidants present in both bark and leaf. The differences observed in studied biological activities in bark and leaf may be ascribed to the differences in composition and concentration of bioactive compounds present in bark and leaf extracts [20, 40].

The present study includes some interesting and important novel findings such as antiamylase, antiglycation, and glycation reversing activity of bark and antiglucosidase, antiglycation, and glycation reversing ability of leaf of authenticated true cinnamon worldwide. Further, this is the first comparative research on bark and leaf of authenticated Ceylon cinnamon for antidiabetic activity (antiamylase and anticholinesterases activities) and its effect on management of diabetic complications (antiglycation and glycation reversing activities). Therefore, important findings on antidiabetic related properties of Ceylon cinnamon would help to enhance the usage among consumers of local and international and it might create a positive financial impact to Sri Lanka as, currently, Ceylon cinnamon is the true cinnamon the world over and the main contributor of the export earnings from spices in the country.

## 5. Conclusions

It is concluded that both bark and leaf of Ceylon cinnamon “true cinnamon” exhibit antidiabetic related properties (mediated via antiamylase and anticholinesterases) and ability to impair development of diabetic complications due to antiglycation and glycation reversing activities. In general, bark showed high antiamylase and antibutyrylcholinesterase activities compared to leaf, whereas leaf showed high antiglycation and glycation reversing activities compared to bark. Thus consumption of Ceylon cinnamon bark and leaf as a dietary supplement may play a vital role in the management of diabetes and its related complications. Further, most importantly findings of this study added value to leaf of Ceylon cinnamon and indicate its potential in developing promising novel antidiabetic food supplements, nutraceuticals, and functional foods and use in adjuvant therapy in the management of diabetes and related complications worldwide.

## Figures and Tables

**Table 1:** Antidiabetic activity of *Cinnamomum* species in vitro.

*Cinnamomum* species	Part used/extract	Activity	References
		*Antiamylase activity *	
*C. zeylanicum* ^*∗*^	Bark aqueous extract	IC_50_: 1.23 ± 0.02 mg/mL	Adisakwattana et al., 2011 [18]
*C. aromaticum *(cassia)^*∗*^	Bark aqueous extract	IC_50_: 1.77± 0.05 mg/mL	Adisakwattana et al., 2011 [18]
*C. loureiroi *(Saigon cinnamon)^*∗*^	Bark aqueous extract	IC_50_: >4.00 mg/mL	Adisakwattana et al., 2011 [18]
*C. zeylanicum (C. verum)* ^*∗*^	Bark hydro alcoholic extract (50 : 50; v/v, water ethanol)	IC_50_: 25 *µ*g/mL	Beejmohun et al., 2014 [19]
*C. verum*	Isopropanol leaf extract	IC_50_: 1 *µ*g/mL	Ponnusamy et al. 2011 [20]
*C. zeylanicum* ^*∗*^	Bark aqueous extract	77% inhibition at 25 mg/mL; 72% inhibition at 12.5 mg/mL and 51% inhibition at 5 mg/mL	Ranilla et al., 2010 [21]

		*Antiglucosidase activity/maltase and sucrase inhibition*	
*C. zeylanicum* ^*∗*^	Bark aqueous extract	100% and 95% inhibition at 2.5 and 0.5 mg/mL, respectively	Ranilla et al., 2010 [21]
*C. zeylanicum* ^*∗*^	Bark aqueous extract	IC_50_ *µ*g/mL: 0.77 ± 0.04 maltase; 0.42 ± 0.02 sucrase	Adisakwattana et al., 2011 [18]
*C. aromaticum *(cassia)^*∗*^	Bark aqueous extract	IC_50_ *µ*g/mL: 0.85 ± 0.04 maltase; 0.88 ± 0.33 sucrase	Adisakwattana et al., 2011 [18]
*C. loureiroi *(Saigon cinnamon)^*∗*^	Bark aqueous extract	IC_50_ *µ*g/mL: 0.96 ± 0.03 maltase; >4.00 sucrase	Adisakwattana et al., 2011 [18]

		*Antiglycation activity*	
Cinnamon (*Cinnamomum* species used not mentioned)^*∗*^	Ethyl acetate and butanol soluble fractions of bark water extract diluted with ethanol	BSA-glucose antiglycation : ethyl acetate soluble fractions: >40% to <90% inhibition at 200 ppm; butanol soluble fractions: <10% to >80% inhibition at 200 ppm BSA-MGO antiglycation: ethyl acetate soluble fractions: <10% to <80% inhibition at 2.5 mg/mL; butanol soluble fractions: <10% to nearly 40% inhibition at 2.5 mg/mL	Peng et al., 2008 [22]
*C. verum* ^*∗*^	Bark methanol extract	BSA-glucose antiglycation: IC_50_: 26 *µ*g/mL	Ho and Chang, 2012 [23]

		*AChE and BChE inhibitory activity*	
*C. zeylanicum*	Bark ethanol extract	AChE: 40.83 ± 0.005 % inhibition at 100 *µ*g/mL; BChE: 51.53 ± 0.005 % inhibition at 100 *µ*g/mL	Kumar et al., 2012 [24]
*C. zeylanicum*	Methanolic leaf extract	IC_50_: AChE 77.78 ± 0.03 *µ*g/mL; BChE 88.62 ± 1.72 *µ*g/mL	Dalai et al., 2014 [25]

^*∗*^Experimental cinnamon sample has no authentication; AChE: acetylcholinesterase; BChE: butyrylcholinesterase.

**Table 2:** Antiamylase activity.

Extract	% inhibition					*µ*g/mL
	Concentration (*µ*g/mL)					
Bark	62.50	125	250	500	1000	IC_50_

Ethanol	24.42 ± 2.22	32.35 ± 1.10	56.91 ± 2.07	74.13 ± 0.53	92.28 ± 1.23	215 ± 10^b^
DCM : M	18.51 ± 0.59	30.20 ± 0.60	57.85 ± 0.47	73.84 ± 2.54	76.19 ± 5.11	214 ± 2^b^

Leaf	93.75	187.50	375	750	1500	IC_50_

Ethanol	4.34 ± 2.37	10.84 ± 1.90	20.78 ± 2.51	43.14 ± 2.46	77.49 ± 2.03	943 ± 28^a^
DCM : M	−4.78 ± 2.06	0.70 ± 2.86	1.81 ± 5.06	9.83 ± 2.91	17.59 ± 1.24	—

Data represented as mean ± SEM (*n* = 4 each). Mean IC_50_ values in the column superscripted by different letters are significantly different at *p* < 0.05. Ethanol bark, DCM : M bark, ethanol leaf, and DCM : M leaf *r*^2^ = 0.99, 1.00, 1.00, and 0.95, respectively. IC_50_: acarbose 133.88 ± 4.4 *µ*g/mL. DCM : M: dichloromethane : methanol.

**Table 3:** Antiglucosidase activity.

Extract	% inhibition
Ethanol bark	−8.11 ± 2.20
DCM : M bark	−5.72 ± 4.89
Ethanol leaf	−8.67 ± 3.19
DCM : M leaf	−7.05 ± 0.86

Data represented as mean ± SEM (*n* = 4 each). % inhibition at 400 *µ*g/mL; IC_50_ acarbose 0.47 ± 0.01 *µ*g/mL.

**Table 4:** Anticholinesterases activity.

	Extract	% inhibition					*µ*g/mL
		Concentration (*µ*g/mL)					
		50	100	200	400	800	IC_50_

Acetylcholinesterase inhibitory activity	Ethanol bark	10.46 ± 2.13	28.68 ± 1.97	30.11 ± 2.37	36.90 ± 2.20	52.13 ± 0.48	804.88 ± 48.69^b^
	DCM : M bark	19.77 ± 2.16	32.34 ± 0.61	37.88 ± 0.11	40.26 ± 0.99	47.69 ± 1.05	966.68 ± 63.18^a^
	Ethanol leaf	6.84 ± 1.44	13.44 ± 2.52	30.08 ± 0.08	35.04 ± 1.59	46.33 ± 3.75	810.96 ± 79.98^a^
	DCM : M leaf	−10.66 ± 3.65	4.03 ± 1.37	14.93 ± 2.31	34.34 ± 1.58	49.13 ± 0.63	879.35 ± 68.00^a^

	Bark	6.25	12.5	25	50	100	IC_50_

Butyrylcholinesterase inhibitory activity	Ethanol	8.50 ± 2.94	17.17 ± 3.69	42.14 ± 3.01	66.06 ± 1.19	75.10 ± 0.75	36.09 ± 0.83^c^
	DCM : M	9.06 ± 2.98	36.79 ± 2.69	50.60 ± 4.42	67.16 ± 0.92	78.97 ± 1.68	26.62 ± 1.66^d^

	Leaf	25	50	100	200	400	IC_50_

Butyrylcholinesterase inhibitory activity	Ethanol	5.42 ± 4.07	25.84 ± 2.20	35.28 ± 1.14	42.62 ± 2.66	49.22 ± 1.81	340.60 ± 18.23^a^
	DCM : M	6.57 ± 2.12	22.95 ± 2.03	37.45 ± 0.38	42.28 ± 0.63	57.48 ± 1.81	261.96 ± 19.56^b^

Data represented as mean ± SEM (*n* = 4 each). Mean IC_50_ values in the column superscripted by different letters are significantly different at *p* < 0.05. Statistical analysis was carried out separately for acetylcholine esterase and butyrylcholine esterase inhibitory assays. IC_50_ galantamine, acetylcholine esterase inhibitory activity: 2.52 ± 0.17 *µ*g/mL; IC_50_ galantamine, butyrylcholine esterase inhibitory activity: 74.80 ± 3.53 *µ*g/mL. Ethanol bark, DCM : M bark, ethanol leaf, and DCM : M leaf *r*^2^ = 0.97, 0.97, 0.94, and 0.98, respectively, for butyrylcholine esterase inhibitory activity. Ethanol bark, DCM : M bark, ethanol leaf, and DCM : M leaf *r*^2^ = 0.92, 0.94, 0.95, and 0.99, respectively, for acetylcholine esterase inhibitory activity. DCM : M: dichloromethane : methanol.

**Table 5:** Total proanthocyanidin content of bark and leaf extracts.

Extract	mg cyanidin equivalents/g of extract
DCM : M bark	1381.53 ± 45.93^a^
Ethanol bark	1097.90 ± 73.01^b^
Ethanol leaf	434.24 ± 14.12^c^
DCM : M leaf	309.52 ± 2.81^d^

Data represented as mean ± SEM (*n* = 6 each). Mean values in the column superscripted by different letters are significantly different at *p* < 0.05.

## References

[1] International Diabetes Federation (2014). *Diabetes Atlas*.

[2] American Diabetes Association (2015). Classification and diagnosis of diabetes. *Diabetes Care*.

[3] Brings S., Fleming T., Freichel M., Muckenthaler M., Herzig S., Nawroth P. (2017). Dicarbonyls and advanced glycation end-products in the development of diabetic complications and targets for intervention. *International Journal of Molecular Sciences*.

[4] Henning C., Glomb M. A. (2016). Pathways of the Maillard reaction under physiological conditions. *Glycoconjugate Journal*.

[5] Nowotny K., Jung T., Höhn A., Weber D., Grune T. (2015). Advanced glycation end products and oxidative stress in type 2 diabetes mellitus. *Biomolecules*.

[6] Sadowska-Bartosz I., Bartosz G. (2015). Prevention of protein glycation by natural compounds. *Molecules*.

[7] Dornadula S., Elango B., Balashanmugam P., Palanisamy R., Kunka Mohanram R. (2015). Pathophysiological insights of methylglyoxal induced type-2 diabetes. *Chemical Research in Toxicology*.

[8] Rabbani N., Xue M., Thornalley P. J. (2016). Dicarbonyls and glyoxalase in disease mechanisms and clinical therapeutics. *Glycoconjugate Journal*.

[9] Ko S. Y., Ko H. A., Chu K. H. (2015). The possible mechanism of Advanced Glycation End Products (AGEs) for Alzheimer's disease. *PLoS ONE*.

[10] Ashford J. W. (2015). Treatment of Alzheimer's disease: the legacy of the cholinergic hypothesis, neuroplasticity, and future directions. *Journal of Alzheimer's Disease*.

[11] De Felice F. G., Lourenco M. V., Ferreira S. T. (2014). How does brain insulin resistance develop in Alzheimer's disease?. *Alzheimer's & Dementia*.

[12] Chawla R., Thakur P., Chowdhry A. (2013). Evidence based herbal drug standardization approach in coping with challenges of holistic management of diabetes: a dreadful lifestyle disorder of 21st century. *Journal of Diabetes Metabolic Disorders*.

[13] Bigliardi B., Galati F. (2013). Innovation trends in the food industry: the case of functional foods. *Trends in Food Science and Technology*.

[14] Padmavathi M. (2013). Chronic disease management with nutraceuticals. *International Journal of Pharmaceutical Science Invention*.

[15] Ranasinghe P., Jayawardana R., Galappaththy P., Constantine G. R., de Vas Gunawardana N., Katulanda P. (2012). Efficacy and safety of ‘true’ cinnamon *(Cinnamomum zeylanicum)* as a pharmaceutical agent in diabetes: a systematic review and meta-analysis. *Diabetic Medicine*.

[16] Abeysekera W. P. K. M., Arachchige S. P. G., Ratnasooriya W. D. (2017). Bark extracts of ceylon cinnamon possess antilipidemic activities and bind bile acids in vitro. *Evidence-Based Complementary and Alternative Medicine*.

[17] Anonymous (2013). *AgStat: Pocket Book of Agricultural Statistics*.

[18] Adisakwattana S., Lerdsuwankij O., Poputtachai U., Minipun A., Suparpprom C. (2011). Inhibitory activity of cinnamon bark species and their combination effect with acarbose against intestinal *α*-glucosidase and pancreatic *α*-amylase. *Plant Foods for Human Nutrition*.

[19] Beejmohun V., Peytavy-Izard M., Mignon C. (2014). Acute effect of Ceylon cinnamon extract on postprandial glycemia: alpha-amylase inhibition, starch tolerance test in rats, and randomized crossover clinical trial in healthy volunteers. *BMC Complementary and Alternative Medicine*.

[20] Ponnusamy S., Ravindran R., Zinjarde S., Bhargava S., Kumar A. R. (2011). Evaluation of traditional Indian antidiabetic medicinal plants for human pancreatic amylase inhibitory effect *in vitro*. *Evidence-Based Complementary and Alternative Medicine*.

[21] Ranilla L. G., Kwon Y.-I., Apostolidis E., Shetty K. (2010). Phenolic compounds, antioxidant activity and *in vitro* inhibitory potential against key enzymes relevant for hyperglycemia and hypertension of commonly used medicinal plants, herbs and spices in Latin America. *Bioresource Technology*.

[22] Peng X., Cheng K.-W., Ma J. (2008). Cinnamon bark proanthocyanidins as reactive carbonyl scavengers to prevent the formation of advanced glycation endproducts. *Journal of Agricultural and Food Chemistry*.

[23] Ho S., Chang P. (2012). Inhibitory effects of several spices on inflammation caused by advanced glycation endproducts. *American Journal of Plant Sciences*.

[24] Kumar S., Brijeshlata, Dixit S. (2012). Screening of traditional indian spices for inhibitory activity of acetylcholinesterase and butyrylcholinesterase enzymes. *International Journal of Pharma and Bio Sciences*.

[25] Dalai M. K., Bhadra S., Chaudhary S. K., Chanda J., Bandyopadhyay A., Mukherjee P. K. (2014). Anticholinesterase activity of *Cinnamomum zeylanicum* L. leaf extract. *Tang [Humanitas Medicine]*.

[26] Anderson R. A., Roussel A. M., Pasupuleti V., Anderson J. W. (2008). Cinnamon, glucose, and insulin sensitivity. *Nutraceuticals, Glycemic Health and Type 2 Diabetes*.

[27] Prasad K. N., Yang B., Dong X. (2009). Flavonoid contents and antioxidant activities from *Cinnamomum* species. *Innovative Food Science and Emerging Technologies*.

[28] Chen P., Sun J., Ford P. (2014). Differentiation of the four major species of cinnamons (*C. burmannii*, *C. verum*, *C. cassia*, and *C. loureiroi*) using a flow injection mass spectrometric (FIMS) fingerprinting method. *Journal of Agricultural and Food Chemistry*.

[29] Ranasinghe P., Perera S., Gunatilake M. (2012). Effects of *Cinnamomum zeylanicum* (Ceylon cinnamon) on blood glucose and lipids in a diabetic and healthy rat model. *Pharmacognosy Research*.

[30] Tailang M., Gupta B. K., Sharma A. (2008). Antidiabetic activity of alcoholic extract of *Cinnamomum zeylanicum* Leaves in alloxon induced diabetic rats. *Peoples Journal of Scientific Research*.

[31] SLSI (2010). *Specification for Ceylon Cinnamon: Sri Lanka Standards*.

[32] Bernfeld P., Colowick S. P., Kaplan N. O. (1955). Amylases, alpha and beta. *Methods in Enzymology*.

[33] Matsui T., Ueda T., Oki T., Sugita K., Terahara N., Matsumoto K. J. (2001). Alpha-glucosidas inhibition isolated anthocyanins. *Journal of Agricultural and Food Chemistry*.

[34] Ellman G. L., Courtney K. D., Andres V., Featherstone R. M. (1961). A new and rapid colorimetric determination of acetylcholinesterase activity. *Biochemical Pharmacology*.

[35] Matsuura N., Aradate T., Sasaki C. (2002). Screening system for the Maillard reaction inhibitor from natural product extracts. *Journal of Health Science*.

[36] Lunceford N., Gugliucci A. (2005). Ilex paraguariensis extracts inhibit AGE formation more efficiently than green tea. *Fitoterapia*.

[37] Premakumara G. A. S., Abeysekera W. K. S. M., Ratnasooriya W. D., Chandrasekharan N. V., Bentota A. P. (2013). Antioxidant, anti-amylase and anti-glycation potential of brans of some Sri Lankan traditional and improved rice (*Oryza sativa* L.) varieties. *Journal of Cereal Science*.

[38] Porter L. J., Hrstich L. N., Chan B. G. (1985). The conversion of procyanidins and prodelphinidins to cyanidin and delphinidin. *Phytochemistry*.

[39] Anonymous (1976). *Ayurveda Pharmacopoeia*.

[40] Abeysekera W. P. K. M., Premakumara G. A. S., Ratnasooriya W. D. (2013). *In vitro* antioxidant properties of bark and leaf extracts of Ceylon Cinnamon (*Cinnamomum zeylanicum* Blume). *Tropical Agricultural Research*.

[41] Singh V. P., Bali A., Singh N., Jaggi A. S. (2014). Advanced glycation end products and diabetic complications. *The Korean Journal of Physiology and Pharmacology*.

[42] Nagai R., Murray D. B., Metz T. O., Baynes J. W. (2012). Chelation: A fundamental mechanism of action of AGE inhibitors, AGE breakers, and other inhibitors of diabetes complications. *Diabetes*.

[43] Bajda M., Wiȩckowska A., Hebda M., Guzior N., Sotriffer C. A., Malawska B. (2013). Structure-based search for new inhibitors of cholinesterases. *International Journal of Molecular Sciences*.

[44] Makhaeva G. F., Lushchekina S. V., Boltneva N. P. (2015). Conjugates of *γ*-Carbolines and phenothiazine as new selective inhibitors of butyrylcholinesterase and blockers of NMDA receptors for Alzheimer disease. *Scientific Reports*.

[45] Zatalia S. R., Sanusi H. (2013). The role of antioxidants in the pathophysiology, complications, and management of diabetes mellitus. *Acta medica Indonesiana*.

[46] Szwajgier D. (2015). Anticholinesterase activity of selected phenolic acids and flavonoids—interaction testing in model solutions. *Annals of Agricultural and Environmental Medicine*.

